# Advances in Murine Models of Diabetic Nephropathy

**DOI:** 10.1155/2013/797548

**Published:** 2013-06-13

**Authors:** Li-li Kong, Hao Wu, Wen-peng Cui, Wen-hua Zhou, Ping Luo, Jing Sun, Hang Yuan, Li-ning Miao

**Affiliations:** Department of Nephrology, The Second Hospital of Jilin University, Changchun 130041, China

## Abstract

Diabetic nephropathy (DN) is one of the microvascular complications of both type 1 and type 2 diabetes, which is also associated with a poor life expectancy of diabetic patients. However, the pathogenesis of DN is still unclear. Thus, it is of great use to establish appropriate animal models of DN for doing research on pathogenesis and developing novel therapeutic strategies. Although a large number of murine models of DN including artificially induced, spontaneous, and genetically engineered (knockout and transgenic) animal models have been developed, none of them develops renal changes sufficiently reflecting those seen in humans. Here we review the identified murine models of DN from the aspects of genetic background, type of diabetes, method of induction, gene deficiency, animal age and gender, kidney histopathology, and phenotypic alterations in the hope of enhancing our comprehension of genetic susceptibility and molecular mechanisms responsible for this disease and providing new clues as to how to choose appropriate animal models of DN.

## 1. Introduction

DN, as one complication of diabetes, is one of the leading causes of end-stage renal disease (ESRD) worldwide. The value of animal models in the study of pathogenesis is beyond doubt. Although great progress has been made in the study of animal models, none of the models can reproduce all the structural and functional changes of human DN. Murine models have substantial advantages over other species in the studies on pathogenesis of DN, including lower cost, murine repositories that bear multiple mutations, plentiful inbred strains, and an available map of murine genomic sequence on the Internet. The Animal Models of Diabetic Complications Consortium (AMDCC) proposes the following three criteria for a desirable murine model of DN: (1) more than 50% decline in glomerular filtration rate (GFR) over the lifetime of the animal; (2) greater than 10-fold increase in albuminuria compared with controls for that strain at the same age and gender; (3) histopathology findings which include mesangial sclerosis (a 50% increase in mesangial volume), any degree of arteriolar hyalinosis, glomerular basement membrane (GBM) thickening (a >25% increase compared with baseline by electron microscopy morphometry), and tubulointerstitial fibrosis. In fact, there are no murine models that meet all of the three criteria. Here we review the identified murine models of DN including artificially induced, spontaneous, and genetically engineered (knockout and transgenic) animal models and compare their advantages and deficiencies. Some of the key issues, such as strain, genetic background, type of diabetes, method of induction, gene deficiency, animal age and gender, kidney histopathology, and phenotypic alterations, are all included in this review. We hope this review could enhance our understanding of genetic susceptibility and molecular mechanisms responsible for DN, as well as provide new clues as to how to choose appropriate animal models of DN. 

## 2. Artificially Induced Murine Models of DN

### 2.1. Murine Models of DN from Type 1 Diabetes Mellitus (T1DM)

Alloxan and streptozotocin (STZ) are widely used for producing artificially induced T1DM which causes kidney damage with similarities to human DN. Both of them are glucose analogues that enter the insulin-producing beta-cells via a glucose transporter. The diabetogenic actions of alloxan and STZ are mediated by reactive oxygen species (ROS). However, they are generated through different approaches in the case of alloxan and STZ. In the presence of glutathione, alloxan and its reduction product, dialuric acid, generate superoxide radicals in a cyclic redox reaction which undergo dismutation to hydrogen peroxide afterwards. Then Fenton reaction ensues with the formation of highly reactive hydroxyl radicals. After being taken into pancreatic beta-cells, STZ is broken down into its glucose and methylnitrosourea moiety. The latter modifies DNA fragments due to its alkylating properties. DNA damage causes the activation of poly ADP-ribosylation which leads to depletion of cellular NAD^+^ and ATP. Enhanced ATP dephosphorylation provides substrate for xanthine oxidase. As a result, superoxide radicals are formed. ROS and a simultaneous cytosolic calcium overload lead to acute necrosis of pancreatic beta-cells [1, 2]. So both of the two diabetogenic agents induce diabetes secondary to the necrosis of pancreatic beta-cells. As time progresses, hyperglycaemia eventually leads to diabetic kidney damage. These STZ-treated models develop a modest degree of proteinuria and serum creatinine increase, as well as minimal mesangial matrix expansion, depending on the genetic background [3, 4]. Animal models of STZ-induced DN are usually performed in mice, Sprague-Dawley and Wistar-Kyoto rats. 

#### 2.1.1. Moderate and High-Dose STZ

Given the mouse strains which are resistant to a single dose of STZ (≥200 mg/kg) or a two-dose regimen of STZ (2 × 100–2 × 125 mg/kg), greater cytotoxicity to pancreatic beta-cells and collateral tissue occurs, resulting in a higher incidence and severity of diabetes [4–7]. Because of the severe hyperglycaemia, the diabetic models need to be monitored for blood glucose and administrated insulin. Several months later, a modest degree of proteinuria can be detected. Some investigators optimize the animal models of STZ-induced DN by uninephrectomy in advance which lead to compensatory hypertrophy of the remaining kidney and acceleration of the disease progression. However, owing to the nephrotoxicity of STZ, it is difficult to differentiate between the direct toxic effect of STZ and lesions caused by hyperglycaemia [8, 9]. Some studies on mouse models of DN show that high-dose STZ-treated mice exhibit more albuminuria than those which receive low-dose STZ, despite of similar blood glucose [10]. Besides, evidence for acute kidney injury caused by high-dose STZ in mice and rats has been reported [9, 11]. Although STZ-treated models result in hypoinsulinemia and hyperglycaemia, they do not share autoimmune features like patients with T1DM. 

#### 2.1.2. Low-Dose STZ

In order to reduce nonspecific nephrotoxicity of STZ, the regimen of multiple low-dose injections of STZ to induce diabetes has been performed, that is daily intraperitoneally injections of 40–60 mg/kg STZ for five consecutive days [4, 12–14] which usually induces repetitive low-grade beta cell damage accompanied by secondary autoimmune insulitis [15, 16]. It is quite different among inbred strains of mice in susceptibility to both pancreatic beta cell toxicity [17] and the direct nephrotoxicity of low-dose STZ [12]. It is reported by Gurley et al. [18] that there is a hierarchical response of blood glucose level to multiple low doses of STZ (DBA/2 > C57BL/6 > MRL/MP > 129/SvEv > BALB/c). His research results also show that males are more susceptible to diabetes induced by this STZ regimen than females. Generally, given strain-appropriate doses of STZ, mice that receive low-dose STZ develop parallel levels of hyperglycemia to those that receive high-dose STZ [9]; the levels of albuminuria, by contrast, are generally lower as a result of reduced direct nephrotoxicity of STZ [19, 20]. Moreover, evidence for nontoxicity on podocytes has been reported. The US-based AMDCC also recommends a standard low-dose model for STZ-induced diabetic complications which include DN. According to this recommendation, mouse models of DN should be induced by daily intraperitoneally injections of 50 mg/kg STZ for five consecutive days. However, studies show that only 50% of C57BL/6 mice develop overt diabetes through this approach. Therefore, whether it will be adopted by relevant experts is not yet determined. 

### 2.2. Murine Models of DN from Type 2 Diabetes Mellitus (T2DM)

The protocol of high-fat diet is widely used to induce insulin resistance and obesity [21–23]. It is also of great use for the research of accelerated atherosclerosis [24–26], although inbred strains of mice exhibit significant differences in response to the effect of high-fat diet. C57BL6 mice respond strongly to high-fat diet; A/J mice, by contrast, are relatively resistant [21]. Sugano et al. [27] have reported a new rat model of DN induced by high-fat diet, multiple low-dose injections of STZ, and uninephrectomy. This model exhibits most features of human DN from T2DM including hyperglycemia, hypoinsulinemia, hyperlipidaemia, hypertension, and microalbuminuria followed by overt albuminuria, mesangial expansion, and terminal glomerular sclerosis. 

## 3. Spontaneous Murine Models of DN

Spontaneous animal models of DN are established by selective breeding from animals which spontaneously develop DN due to genetic abnormality. Renal abnormalities in these models resemble human diseases; therefore, these models provide an experimental platform for studying pathogenesis and genetic susceptibility responsible for DN. Although these models are difficult to feed and breed, not widely available, and with long modeling cycle and higher cost, the application of them is becoming increasingly extensive.  Table 1 lists some common murine models of spontaneous DN.

### 3.1. Murine Models of Spontaneous DN from T1DM

#### 3.1.1. Nonobese Diabetic (NOD) Mouse

The spontaneous murine model of T1DM that has been studied most extensively is the NOD mouse. Due to pathogenic and genetic similarities to the human disease, the model serves as a useful tool to study the etiology, pathology, and progression of disease. NOD mouse was derived from the Jcl: ICR cataract mouse 30 years ago in Japan [28]. These mice develop spontaneous insulitis at the age of 4-5 weeks, and overt diabetes emerges at the age of 24–30 weeks when most of pancreatic *β*-cells are destroyed. Female incidence of diabetes is four times higher than the male in NOD mice [10]. This model exhibits a number of clinical features of human T1DM including hyperglycaemia, glycosuria, polyuria, and polydipsia; however, it is more resistant to ketoacidosis. Without insulin administration, NOD mice usually die of dehydration, rather than ketoacidosis. Like in humans, the major histocompatibility complex (MHC) alleles are closely related to susceptibility to T1DM. Moreover, some MHC alleles must be accompanied by other non-MHC genes for the development of T1DM [29, 30], which is also the same in human disease. Even so, NOD mice are not the perfect animal model for human T1DM. As an inbred strain, they have a fixed genetic risk for T1DM and develop T1DM in a predictable fashion [31], whereas human T1DM develops as a result of deleterious interactions between relevant genes and from external environmental factors. 

 Despite of the extensive study of the genetic and immunologic pathogenesis of T1DM in NOD mouse, few investigators choose NOD mouse to do research on DN because of the complicated genetics, the late and variable age of onset of T1DM, and the requirement for insulin administration [10]. Even so, one of the few studies shows that the amount of albuminuria in hyperglycemic NOD mice is seven times higher than that in NOD mice before the development of hyperglycemia [32]. The results of renal lesions in acute phase of T1DM in NOD mice show mild changes in glomeruli and structural alteration of the proximal straight tubules. The study also points out that increased neuronal nitric oxide synthase may represent one of the pathogenic factors of DN [70]. Besides, there are also studies using NOD mice demonstrating that transforming growth factor-*β* (TGF-*β*) and advanced glycosylation endproducts (AGE) take an important role in mesangial proliferation and sclerosis [71–73], which is also the same in human DN. 

#### 3.1.2. Insulin-2 Akita Mouse

Akita mice develop T1DM because of the spontaneous mutation in Ins-2 gene. The mutation leads to the misfolding of insulin protein, which is toxic to pancreatic *β*-cell. Consequently, the capacity of *β*-cell to secrete insulin is largely decreased. The Ins-2 Akita mutation which is autosomal dominant was originally found in C57BL/6 mice in Akita, Japan. Mice heterozygous for the mutation develop significant hyperglycemia as a result of severe insulin deficiency at 3 to 4 weeks of age, but homozygotus mice usually die in perinatal period. Males develop substantially worse insulin deficiency than females. Mice with Ins-2 Akita mutation exhibit modest levels of albuminuria and mild-to-moderate glomerular mesangial expansion [18]. Nonetheless, Akita mice have higher levels of hyperglycemia, albuminuria, blood pressure, and more consistent structural changes of kidney compared with STZ-induced DN [18]. Gurley et al. have found that renal phenotype of Akita mice is largely dependent on their genetic background strains [18]. It means that genetic factors might influence susceptibility to DN in Akita mice which is the same in human disease. Thus, mice bearing Ins-2 Akita mutation have significant advantages as a model of T1DM. 

### 3.2. Murine Models of Spontaneous DN from T2DM

#### 3.2.1. *Db/db* Mouse

The *db/db* mouse which has a G-to-T mutation in the gene coding the leptin receptor develops obesity, insulin resistance, and T2DM spontaneously. The *db/db* mutation which is autosomal recessive was initially recognized from an obese and hyperphagic mouse in the C57BLKS/J strain and was subsequently backcrossed to a pure C57BL/6J background. Mice in the C57BLKS/J stain exhibit hyperinsulinemia at 10 days of age and slight hyperglycemia at 1 month of age. Overt hyperglycemia is noted by 8 weeks of age [20]. Manifestations of DN are albuminuria, glomerular hypertrophy, mesangial matrix expansion, and GBM thickening [20]. Albuminuria can be detected as early as 3 to 4 weeks after the onset of hyperglycemia [3]. The level of albuminuria in the *db/db* male mouse is 68–600 *μ*g/24 h [20, 34–37] which is only 4–21 *μ*g/24 h [34, 37] in the age-matched heterozygous littermate. The *db/db* mice display an increase in glomerular size and mesangial matrix by 5-6 months of age [20]. By 18–20 months, the glomerular and mesangial matrix enlargements become more remarkable, and thickening of the GBM is observed [20]. In general, *db/db* mice do not develop mesangiolysis, nodular mesangial sclerosis, and progressive renal insufficiency [33]. However, they are good models of early changes in human DN. Hyperglycemia, and renal changes of *db/db* mice in C57BLKS/J stain are usually worse than in the C57BL/6 background. Hence, investigations of DN in *db/db* mice are more widely conducted with the C57BLKS/J strain. Nonetheless, *db/db* mice in the C57BL/6 stain which have been intercrossed with gene knockout and transgenic mice [74, 75] provide new strains to identify pathogenesis of DN.

#### 3.2.2. Otsuka Long-Evans Tokushima Fatty (OLETF) Rat

The OLETF rat is an identified murine model of T2DM. The model is characterized by hyperphagia, mild obesity, late-onset hyperglycemia, hypertension, dyslipidemia and advanced DN. Multiple recessive genes are associated with the induction of diabetes, including *odb-1* on X-chromosome of OLETF rats. It is also reported that a major quantitative trait locus colocalizing with cholecystokinin type A receptor gene influences poor pancreatic proliferation in OLETF rats [38]. The progression of T2DM in OLETF rats can be prevented by exercise [76] and calorie-restricted diet [77] as human disease. At 12–20 weeks of age, OLETF rats exhibit mild obesity and hyperinsulinemia [39]. Late-onset hyperglycemia is noted by 18 weeks of age [39]. At 22 weeks of age, the OLETF rats develop overt albuminuria, and at 54 weeks, advanced renal changes such as macroalbuminuria, nodular lesions, diffuse glomerulosclerosis, and tubulointerstitial fibrosis are noted like human DN [40]. Therefore, OLETF rat is considered as one of the best murine models to study DN.

#### 3.2.3. GK Rat

Goto Kakizaki (GK) rat, a spontaneous polygenic model of T2DM, is established by repeated inbreeding of glucose-intolerant Wistar rats over many generations [78]. This model is characterized by moderate hyperglycemia, peripheral insulin resistance, and nonhyperlipidemia, and nonobese phenotype. It is recognized that T2DM in GK rat is primarily caused by beta-cell deficit. In order to identify the origin of the abnormality, Miralles and Porthe [79] compared the development of the embryo in GK and Wistar rats. They found a decrease in pancreatic cell proliferation from embryonic day 16 to 20 (E16–E20) and a wave of pancreatic cell apoptosis from E16 to E18. By E16, the number of pancreatic beta-cells in the GK rats is half of the Wistar rats, and this difference was sustained until birth. GK rat exhibits morphological changes which can be seen in early stage of human DN such as glomerular hypertrophy and GBM thickening [41]. It does not develop overt proteinuria or progressive nephropathy by 8 months of age [41], even being treated with some initiators to promote renal injury [42, 43]. However, Sato et al. [44] have reported advanced renal changes in GK rats such as segmental glomerulosclerosis and tubulointerstitial fibrosis at 24 months of age. At that time, albuminuria increases notably. Thus, they draw the conclusion that renal changes in GK rats at a late stage were similar to those of progressive human DN. Therefore, GK rat serves a useful tool for studying T2DM and DN.

#### 3.2.4. *Ob/ob* and BTBR *ob/ob* Mice

Compared with *db/db* mouse, *ob/ob* mouse develops T2DM caused by the spontaneous recessive mutation in leptin [46], the ligand for the leptin receptor. The *ob/ob* mutation exists in C57BL/6J, DBA2/J, and FVB strains. This model exhibits only mild functional and morphological changes in C57BL/6J strains [80]. Thus, it is not widely used as an animal model of DN. 

 However, a new mouse model that mimics progressive DN has been developed in BTBR strain with the *ob/ob* mutation [47]. Its characteristics are insulin resistance, hyperinsulinemia, pancreatic islet hypertrophy, severe hyperglycemia, obesity, hypercholesterolemia, and elevated triglycerides. The BTBR *ob/ob* mice are largely resistant to the hypoglycemic effect of insulin administration and rapidly develop pathological changes of both early and advanced human DN [48]. The mice develop progressive proteinuria by 4 weeks of age. Characteristics of early DN such as glomerular hypertrophy, accumulation of mesangial matrix, and loss of podocytes are detectable by 8 weeks of age [48]. Glomerular lesions of progressive, advanced DN are present by 20 weeks. By 22 weeks, morphological characteristics of renal injury has 20% increase in GBM thickness, 50% increase in mesangial matrix, mesangiolysis, diffuse mesangial sclerosis, focal arteriolar hyalinosis, and focal mild interstitial fibrosis [48]. On the one hand, the advantage of BTBR *ob/ob* mice over other animal models is the relatively short period for the development of advanced DN. On the other hand, compared with *db/db* mice (with deficiency of the leptin receptor), BTBR *ob/ob* mice provide a new tool for testing therapeutic effect of leptin administration in DN, whereas the mice have apparent limitations, such as their high cost and infertility [33]. Besides, due to the limited study on BTBR strain, investigators have to pay great attention to the time-consuming backcrossing strategies in order to induce specific genetic mutations into these mice [33]. Nonetheless, BTBR *ob/ob* mouse is fairly valuable for testing therapeutic interventions. 

#### 3.2.5. New Zealand Obese (NZO) Mouse

The NZO mouse is established by selective breeding from polygenic mice of obesity and T2DM in New Zealand. It is characterized by obesity, T2DM, and low-titer IgM antibodies to the insulin receptor [49]. The QTLs on chromosomes 1, 2, 4, 5, 6, 7, 11, 12, 13, 15, 17, and 18 [81–83] are responsible for the disease. NZO mice are prone to autoimmune disease and develop circulating antibodies to both native DNA and single-stranded DNA [49]. By 6 months of age, the antibody levels in NZO mice are comparable to those found in the mouse models of systemic lupus erythematosus [49]. NZO mice exhibit morphological features of both diabetic and lupus nephropathies, such as glomerular proliferation, mesangial deposits, mild GBM thickening, glomerulosclerosis, eosinophilic nodules in some glomeruli, occasional hyalinization of the glomerular arterioles, and healing arteriolar inflammation [49]. Thus, given the evidence of immune disorder, NZO mouse offers a unique opportunity to study the relationship among T2DM, autoimmunity, and obesity.

#### 3.2.6. KK-Ay Mouse

KK-Ay mice develop T2DM caused by the dominant mutation in agouti yellow (Ay) gene. The Ay gene is expressed in the hair follicle, where the gene product acts as an antagonist of melanocyte stimulating hormone receptor resulting in the inhibition of melanogenesis and yellow fur [50]. Besides, an agouti-related protein influences weight regulation [51]. It is widely recognized that Ay gene is crucial to the yellow obese phenotype. KK-Ay mice spontaneously exhibit severe, early-onset hyperinsulinemia, hyperglycemia, obesity, hypertriglyceridemia, fatty liver, and albuminuria. KK-Ay mice develop morphological changes of early DN, such as glomerular hypertrophy, mild and moderated mesangial matrix expansion, and segmental proliferative glomerular nephritis [52]. Therefore, KK-Ay mouse might be a useful murine model of the early stage of DN. It is notable, though, that most of male KK-Ay mice die of obstructive uropathy associated with hydronephrosis between 7 and 14 months of age without unknown causes [53]. Partly for this reason, KK-Ay mouse is not widely used as animal model of DN.

#### 3.2.7. Zucker Diabetic Fatty (ZDF) Rat

The ZDF rat which has a missense mutation in the gene coding the leptin receptor (fa/fa) [54] spontaneously develops insulin resistance, T2DM, hyperlipidemia, both moderate hypertension and obesity, and progressive renal injury. Studies of the ZDF rats show that the hyperglycemia is sexually dimorphic. Although female ZDF rats have similar levels of insulin resistance and degrees of obesity to male ZDF rats, they develop hyperglycaemia only when administered with diabetogenic diet [84]. Thus, the ZDF male rats are more widely used as the animal models of T2DM and DN. Histopathologic changes of kidney [55–60] have been described as focal segmental glomerulosclerosis, mild mesangial expansion, macrophage infiltration, and interstitial fibrosis. Hyperglycaemia in ZDF rats is manifested by 12 weeks [61]. These rats develop albuminuria at 14 weeks [62] and focal segmental glomerulosclerosis at 18–20 weeks of age [56, 57]. Macroalbuminuria ultimately leads to chronic renal insufficiency by 22 weeks of age [62]. The ZDF rat exhibits a physiological and metabolic profile similar to human T2DM and functional and morphological renal lesions that resemble human DN. Thus, ZDF rat is considered as an excellent animal model of T2DM and DN.

## 4. Genetically Engineered (Knockout and Transgenic) Murine Models of DN

Diabetes is the major cause of ESRD worldwide. Despite of the high incidence, only a minority of patients with diabetes develop renal lesions. Family-based studies show that a significant genetic component confers risk for DN. All the above studies indicate the importance of genetic factors in differential susceptibility to DN, whereas limited progress has been made in identifying specific genetic factors that contribute to DN due to genetic heterogeneity and multigenic pathogenesis. Recently, investigators have developed genetically engineered murine models in combination with genetic manipulations, including transgenic and knockout mice bearing defined alterations in a single gene or in a series of candidate genes. Studies *in vivo* using these models show that the genes coding TGF-*β*, plasma prorenin, inducible cAMP early repressor, receptor for advanced glycation endproducts (RAGE), endothelial nitric oxide synthase (eNOS), and aldose reductase involve in the origin and progression of DN, corroborating experimental findings from human association studies. Genetically engineered murine models provide valuable insight into the role of pathogenetic genes and molecular mechanisms responsible for DN, thus opening new avenues to develop novel therapeutic strategies.  Table 2 lists some of the genetically engineered murine models that develop advanced DN-like human disease. Here we emphasize more on the OVE26 and the eNOS^−/−^/*db/db* mice, of type 1 and type 2 diabetes, that develop kidney injury most resembling that seen in human. 

### 4.1. OVE26 Mouse

The OVE26 mouse on the FVB inbred strain is a transgenic mouse model of severe early-onset type 1 diabetes [63]. These mice exhibit severe hyperglycemia 2-3 weeks after birth due to *β*-cell-specific damage in response to overexpression of calmodulin transgene regulated by the insulin promoter. Zheng et al. has reported progressively increasing albuminuria which is 305 *μ*g/24 h by 2 months and 15,000 *μ*g/24 h by 9 months of age [64]. They also detect hypoalbuminemia, increased GFR from 2-3 months, and the following decreased GFR from 5–9 months, as well as hypertension which coincided with increasing albuminuria. The OVE26 mice develop morphological changes of advanced DN including enlarged glomeruli, enlarging mesangium with diffuse and nodular expansion of mesangial matrix, GBM thickening, diffuse and nodular glomerulosclerosis, nodules similar to typical K-W nodules, expansion of the tubules, atrophy of tubular cells, interstitial infiltration of mononuclear cells, and tubulointerstitial fibrosis. A low level of pancreatic beta cell survival allows OVE26 mice to survive well over a year with no insulin treatment and maintain near normal body weight. The functional and morphological changes in OVE26 mice closely resemble human DN. Therefore, OVE26 mice provide a valuable model of advanced human DN. However, severe albuminuria in OVE26 mice highly depends on FVB background. Albuminuria, mesangial matrix expansion, and fibrosis are all significantly diminished when OVE26 mice were crossbred with C57BL6 or DBA2 mice [65]. This strain dependence makes it difficult to introduce other genetic mutations from other backgrounds into this model.

### 4.2. eNOS^−/−^/*db/db* Mouse

The eNOS^−/−^/*db/db* mouse is a model of type 2 diabetes generated by backcrossing of eNOS knockout mouse on the C57/B6 background with *db/db* mouse on the C57BLKS/J (BKS) background [66]. The eNOS^−/−^/lepr^*db/db*^ double-knockout mice exhibit obesity, hyperglycemia, hyperinsulinemia, hypertension, dramatic albuminuria, and decreased GFR [67, 68]. These mice develop histopathological changes of DN-like human disease such as mesangial expansion, GBM thickening, mesangiolysis, focal segmental and nodular glomerulosclerosis, nodules that resemble K-W nodules, striking fibronectin accumulation in glomeruli, arteriolar hyalinosis, minimal tubulointerstitial fibrosis, and microaneurysms. These features establish this mouse model as one of the very few to develop features of advanced DN.

## 5. Conclusions

With increasing incidence of DN, development of an ideal animal model becomes one of the top priorities in combating this health crisis. Murine models have significant advantages over other species in pathogenesis investigation. Impact of genetic background on several murine models of DN is evident on the susceptibility to diabetes-associated renal injury, the severity and histopathology of renal lesions. STZ-treated mice and rats are widely used as animal models of early DN due to their cost effectiveness and the absence of advanced pathological lesions. Some mutations in spontaneous murine models enhance our understanding of pathophysiological mechanisms of DN. Genetic engineering enables us to insert or delete a specific gene or a series of candidate genes, providing valuable insight into the role of pathogenetic genes and molecular mechanisms responsible for DN, as well as opening new avenues to develop novel therapeutic strategies. However, it is difficult to establish an animal model that recapitulates all the features of human DN. Few models develop morphologically advanced DN; among these, OLETF rats, OVE26 mice, BTBR *ob/ob* mice, eNOS^−/−^/*db/db* mice, RAGE/iNOS mice, and megsin/RAGE/iNOS mice seem to be the most robust. Perhaps the last four models suggest to us that more robust murine models of DN can be established by superimposed genetic mutations or crossbreeding with an entirely different strain. Novel animal models that reproduce human DN have yet to be established in the future.

## Figures and Tables

**Table 1:** Murine models of spontaneous DN.

Models (Ref)	Strains	Defects	Phenotypic alterations	Kidney pathology
NOD mouse [10, 28–32]	Inbred line derived from ICR (outbred line)	Autoimmune insulitis caused by polygenes including specific MHC class II alleles and many non-MHC loci	T1DM, hyperglycemia, albuminuria, autoimmune insulitis, and other autoimmune manifestations	Related reports are few, enlarged glomeruli, and mesangial sclerosis

Insulin-2 Akita mouse [10, 18, 33]	C57BL/6, C3H	Autosomal dominant mutation in the Ins-2 gene causes misfolding of insulin protein	T1DM, hyperglycemia, and modest levels of albuminuria	Increased mesangial matrix, GBM thickening, and no mesangiolysis or widespread marked or nodular mesangial sclerosis

*db/db* mouse [10, 20, 33–37]	C57BL/6, C57BLKS, DBA, FVB, CBA	G-to-T mutation in the gene coding the leptin receptor (*db/db*)	T2DM, hyperglycemia, obesity,and albuminuria	Glomerular hypertrophy, mesangial matrix expansion, GBM thickening, and no mesangiolysis or nodular mesangial sclerosis

OLETF rat [38–40]	Long-Evans	Poor pancreatic proliferation caused by multiple genes including several QTLs and the gene encoding CCKAR	T2DM, mild obesity, late-onset hyperglycemia, macroalbuminuria, hypertension, and dyslipidemia	Glomerular hypertrophy, GBM thickening, extracellular matrix expansion, nodular lesions, diffuse glomerulosclerosis, and severe tubulointerstitial fibrosis

GK rat [41–45]	Wistar	Pancreatic beta-cell deficit caused by polygenes	T2DM, hypertension, moderate hyperglycemia, albuminuria, nonobesity, nonhyperlipidemia	Glomerular hypertrophy and GBM thickening by 35 weeks; segmental glomerulosclerosis and tubulointerstitial fibrosis at 24 months of age

BTBR *ob/ob* mouse [46–48]	BTBR	*ob/ob * mutation (a recessive mutation in the gene coding leptin) in BTBR strain	T2DM, severe hyperglycemia, pancreatic islet hypertrophy, macroalbuminuria, hypercholesterolemia, elevated triglycerides, obesity, and decreased GFR	Glomerular hypertrophy, mesangial matrix expansion, GBM thickening, loss of podocytes, diffuse mesangial sclerosis (focally approaching nodular glomerulosclerosis), focal mild interstitial fibrosis, focal arteriolar hyalinosis, and mesangiolysis

NZO mouse [10, 49]	NZO	Obesity/diabetes caused by polygenes including QTLs on chromosomes 1, 2, 4, 5, 6, 7, 11, 12, 13, 15, 17, and 18	T2DM, obesity, hyperglycemia, albuminuria, low-titer IgM antibodies to the insulin receptor, and susceptibility to lupus nephritis	Glomerular proliferation, mesangial deposits, mild GBM thickening, glomerulosclerosis, eosinophilic nodules in some glomeruli, occasional hyalinization of the glomerular arterioles, and healing arteriolar inflammation

KK-Ay mouse [10, 50–53]	KK, C57BL, C3H, FVB	Yellow/obese/diabetic phenotype caused by polygenes including dominant mutation in agouti yellow (Ay) gene	T2DM, hyperglycemia, obesity, albuminuria, hypertriglyceridemia, and obstructive uropathy	Glomerular hypertrophy, mild and moderated mesangial matrix expansion, and segmental proliferative glomerular nephritis

ZDF rat [54–62]	Zuker	Missense mutation in the gene coding the leptin receptor (*fa/fa*)	T2DM, hyperglycemia, obesity, hyperlipidemia, hypertension, and macroalbuminuria	mesangial expansion, focal segmental glomerulosclerosis, macrophage infiltration, and interstitial fibrosis

CCKAR: cholecystokinin type A receptor; GFR: glomerular filtration rate; GBM: glomerular basement membrane; MHC: major histocompatibility complex; QTLs: quantitative trait loci.

**Table 2:** Genetically engineered murine models of DN.

Models (Ref)	Strains	Molecular background	Phenotypic alterations	Kidney pathology
OVE26 mice [63–65]	FVB	*β*-cell-specific damage due to overexpression of calmodulin transgene regulated by the insulin promoter	T1DM, hyperglycemia, hypertension, albuminuria, hypoalbuminemia, and GFR increased first and then decreased	Enlarged glomeruli, enlarging mesangium with diffuse and nodular expansion of mesangial matrix, GBM thickening, diffuse and nodular glomerulosclerosis, nodules similar to typical K-W nodules, expansion of the tubules, atrophy of tubular cells, interstitial infiltration of mononuclear cells, tubulointerstitial fibrosis

eNOS^−/−^/*db/db* mice [66–68]	C57/B6 × C57BLKS/J (BKS)	BKS-*db/db* mice cross with eNOS^−/−^ mice	T2DM, obesity, hyperglycemia, hypertension, albuminuria, and decreased GFR	Mesangial expansion, GBM thickening, arteriolar hyalinosis, mesangiolysis, microaneurysms, focal segmental and nodular glomerulosclerosis, nodules that resemble K-W nodules, striking fibronectin accumulation in glomeruli, minimal tubulointerstitial fibrosis

RAGE/iNOS mice [45]	CD-1	Transgenic mice that overexpress human RAGE in vascular cells crossbreed with another transgenic line carrying human cDNA for iNOS under the control of the insulin promoter	T1DM, hyperglycemia, albuminuria, and increased serum creatinine	Mesangial expansion, diffuse glomerulosclerosis

Megsin/RAGE/iNOS mice [69]	C57BL/6N × CD1	Triple transgenic mice overexpressing megsin, RAGE, and iNOS (megsin transgenic mice crossbreed with RAGE/iNOS transgenic mice)	T1DM, hyperglycemia, albuminuria, increased serum creatinine, and urea nitrogen	Glomerular hypertrophy, diffuse mesangial expansion, GBM thickening, global mesangial sclerosis, some segmental sclerotic lesions that resemble K-W nodules, inflammatory cell infiltration, interstitial fibrosis, and immune complexes depositions

eNOS: endothelial nitric oxide synthase; GBM: glomerular basement membrane; GFR: glomerular filtration rate; iNOS: inducible nitric oxide synthase; K-W nodules: Kimmelstiel-Wilson nodules; RAGE: the receptor for advanced glycation endproducts.
